# Decoding urbanization pathways: An integrative land use change indicator framework for sustainable development in Nusantara

**DOI:** 10.1016/j.isci.2026.116763

**Published:** 2026-07-09

**Authors:** Achmad Ghozali, Walter Timo de Vries

**Affiliations:** 1 Chair of Land Management, School of Engineering and Design, Technical University of Munich, Munich, Germany; 2 Urban and Regional Planning, Institut Teknologi Kalimantan, Balikpapan, Indonesia

**Keywords:** LUC, Nusantara, predictive-preventive framework, sustainable development, urbanization pathways

## Abstract

Indonesia’s new capital, Nusantara, is generating urban expansion that extends beyond administrative boundaries, yet its cross-boundary land-use dynamics remain insufficiently understood. This study develops a contextualized predictive-preventive framework of land use change (LUC) indicators for future urbanization assessment in the Nusantara agglomeration through a review-based conceptual synthesis. Drawing on recent LUC studies, published empirical evidence, and the Nusantara Development Masterplan, the study identifies 46 predictive indicators representing internal urban growth conditions and six preventive indicators capturing regional ecological capacity and spatial inequality. Predictive indicators explain land-conversion suitability and growth pressure, whereas preventive indicators function as safeguards that regulate or moderate expansion in environmentally fragile or unequal areas. The framework links internal urban drivers with regional socio-ecological constraints, extending LUC assessment beyond administrative borders. It provides a conceptual foundation for future scenario modeling, comparative research, and sustainable land management in new city development contexts.

## Graphical abstract

**Figure:**
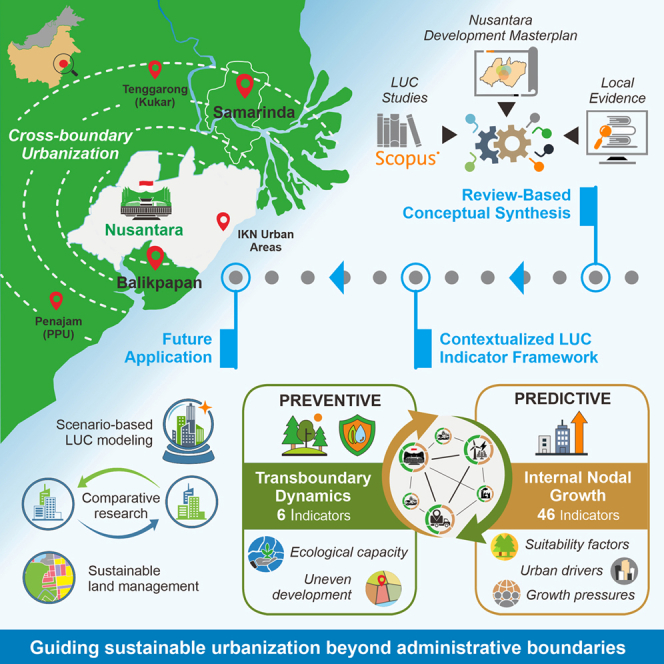

## Teaser

Geography; Environmental science

## Introduction

New city developments (NCDs), as opposed to the redevelopment of existing ones, are increasingly adopted in developing countries as strategies to address spatial, demographic, and environmental pressures. Beyond these functional motives, such projects are often driven by symbolic goals of modernization and national identity.^1^ Indonesia’s relocation of its capital from Jakarta to Nusantara (also known as IKN) in East Kalimantan epitomizes both ambitions. Announced in 2019, the project aims to mitigate Jakarta’s socioeconomic and environmental crises by establishing a green, smart, and inclusive urban centre supported by sustainable and efficient infrastructure.^2^ Projected to house 1.9 million residents by 2045, the city functions as a new growth pole intended to stimulate economic multiplier effects across surrounding municipalities.^3^ Given its regional-scale development, examining Nusantara’s integration of space, demographics, and ecology offers critical insights into contemporary urban transformation.

A critical lens for studying these processes involves evaluating and predicting land use change (LUC). Establishing Nusantara entails the extensive conversion of natural ecosystems into built environments and infrastructure, fundamentally reshaping the physical and social landscapes of existing communities and surrounding regions.^4^ Moreover, its integration with Balikpapan and Samarinda positions the region as a unified economic triangle, strengthening value chains and stimulating spillover effects beyond administrative boundaries.^5^ Paradoxically, these dynamics might risk replicating the environmental and socio-demographic crises of the Jakarta region, potentially threatening biodiversity and local livelihoods in Kalimantan.^6^

Despite growing interest in Nusantara’s development and future spatial dynamics, most studies focus exclusively on processes within its administrative boundaries. While prior research has examined socioeconomic and environmental dimensions,^5^^,^^7^ cross-boundary urban interactions and regional urbanization dynamics remain underexplored. Since land transformation drivers in new cities are inherently uncertain and context-specific,^8^ this localized focus has resulted in a gap regarding regional spatial development and sustainable land management strategies in current academic discourse.

Decoding future urbanization is crucial for managing development complexities while balancing environmental sustainability with socioeconomic advancement. However, conventional narratives often reduce LUC to a mere consequence of urban growth, overlooking its role in shaping urban form. Although scholars acknowledge that physical geography, climate, socioeconomics, and infrastructure dictate urban trajectories, there is no consensus on how these factors interact or which exert the most decisive influence. Factor selection in LUC modeling frequently prioritizes data availability or methodological convenience over systematic frameworks,^9^ undermining predictive reliability and policy relevance. Therefore, examining how LUC factors operate within specific local contexts is fundamental to developing robust modeling frameworks and improving land-use management.

In response, this study develops an integrative framework for assessing future urbanization and its challenges. By synthesizing indicators from contemporary LUC research with a contextual evaluation of the Nusantara Development Masterplan, we propose a conceptual model that transcends administrative boundaries to capture regional interdependence. This study addresses two key questions: (RQ1) Which spatial driving forces in current LUC research are pertinent to predicting urban expansion in the Nusantara region? and (RQ2) How does the Nusantara Development Masterplan influence regional spatial dynamics and address emerging urban challenges? Adopting a review-based conceptual synthesis grounded in literature review and document analysis, this article establishes a contextualized LUC indicator framework tailored for the Nusantara agglomeration.

The remainder of this paper is organized as follows. The Literature overview section reviews existing research on Nusantara’s design and development. The method details section presents the methodological framework for retrieving and categorizing empirical studies on predictive and preventive measures of urban expansion. The Results section synthesizes and aligns predictive indicators with Nusantara’s spatial strategies. Finally, the Discussion section interprets the role of preventive indicators, proposes a unified LUC framework, and outlines a research agenda for assessing future urbanization in the Nusantara agglomeration.

## Literature overview

### Forest-sponge-smart spatial vision for Nusantara development

In accordance with Indonesian Presidential Regulation No. 63 of 2022, Nusantara’s development structure comprises three interlinked concepts—Forest City, Sponge City, and Smart City. This vision seeks to reconcile high-density urbanization with ecological preservation, climate resilience, and digital innovation.^2^

The Forest City strategy prioritizes ecological integrity by curbing horizontal expansion and retaining at least 65% of the administrative area as tropical rainforest, mangroves, and conservation zones. In spatial terms, this necessitates strict zoning to confine built-up expansion to designated clusters and maintain contiguous forest patches and mangrove areas. However, recent spatiotemporal analyses reveal declining regional biomass and rising land surface temperatures,^10^ suggesting that current Nusantara development trajectories already threaten forest retention targets. Consequently, strict adherence to these regulations is considered the only viable scenario for preserving East Kalimantan’s ecosystem services, underscoring the necessity of robust preventive controls on land conversion.^11^

The Sponge City pillar, inextricably linked to the forest concept, integrates hydrological resilience into urban design, enabling the landscape to absorb, store, and purify runoff. This vision addresses recent projections of a significant increase in heavy precipitation days within the Nusantara region by the late century.^12^ Accordingly, scholars prioritize nature-based solutions (NbSs) as the primary mechanism to achieve the Zero Delta Q principle. Specifically, Yuanita et al.^13^ highlight the role of riparian forests and floodplains in mitigating hydrometeorological risks, while Prihanto et al.^14^ argue that seasonal water scarcity necessitates large-scale rainwater harvesting to ensure long-term water security for the growing population. The Sponge concept involves reserving land for retention basins, riparian buffers, and permeable open spaces, which inherently constrains the expansion of impervious surfaces and high-density development. Consequently, water-sensitive planning serves as a vital preventive mechanism for maintaining flood control, groundwater recharge, and thermal comfort amidst rapid urbanization.

Complementing these ecological strategies, the Smart City layer functions as an integrative mechanism through digital governance and infrastructure. The Nusantara Masterplan organizes this agenda into six clusters, including security, digital governance, environmental monitoring, mobility, and livability.^15^ From a land-use perspective, smart governance aims to operationalize real-time monitoring of land conversion, digitized permitting, and data-driven enforcement of zoning standards. However, empirical studies highlight systemic deficits in human capital, fiscal capacity, and bureaucratic agility that could impede implementation.^16^ Sensuse et al.^17^ therefore advocate for a “smart bureaucracy” to embed these technologies into governance workflows. Simultaneously, energy modeling indicates that a hybrid renewable system—combining solar PV, natural gas, and storage—is essential to achieve the Net Zero 2045 vision.^18^ These findings expose a critical implementation gap: Smart visions may remain superficial if physical development outpaces institutional capacity and regional coordination.

Across these thematic strands, the Nusantara Masterplan integrates the Forest, Sponge, and Smart City concepts into a unified framework designed to deliver a “World City for All” and fulfill the Net Zero 2045 commitment.^19^ In this vision, green-blue corridors and digital monitoring systems serve as the primary spatial mechanisms linking vegetation, hydrological networks, and urban infrastructure. High-level concepts such as smart forestry, nature-based economies, and digital environmental resilience frame how ecological protection and technological innovation should reinforce one another.^15^ Operationally, the Masterplan translates these ideals into a diverse array of physical and digital infrastructures—ranging from conservation zones and sponge parks to advanced ICT backbones—as detailed in Table S1.

**Figure 1:**
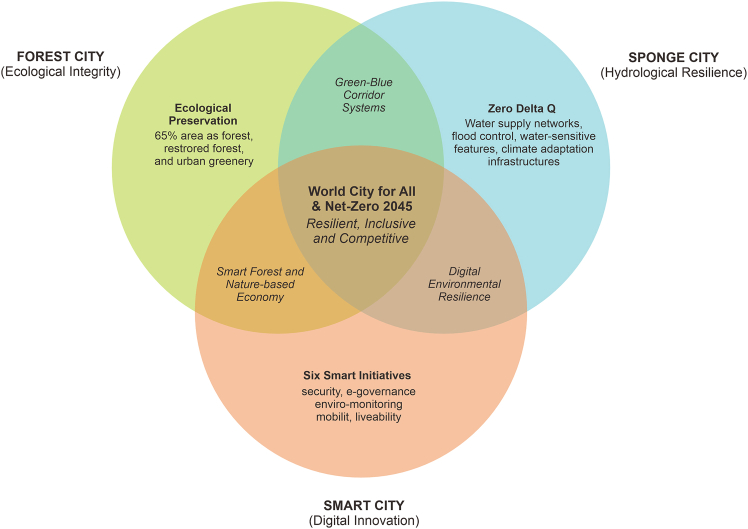
Forest-Sponge-Smart integration framework in the Nusantara masterplan (Green) The Forest City, (Blue) Sponge City, and (Red) Smart City principles are integrated into overlapping strategies which then translate the core vision of Nusantara development as World City for All.

These visions imply a set of critical spatial indicators—including forest-retention ratios, the continuity of green-blue corridors, flood exposure under extreme rainfall, energy-emission intensity, and smart services—that are essential for evaluating whether Nusantara’s ambitions can withstand rapid urban expansion. However, the extent to which these integrated concepts have been translated into enforceable land-use instruments remains weakly evidenced. Furthermore, few studies assess how these strategies will perform under actual land conversion and regional development pressures. Against this background, Figure 1 synthesizes these planning ideals into a conceptual Forest-Sponge-Smart framework that underpins the predictive-preventive indicator system used to analyze LUC and sustainability trade-offs in the Nusantara region.

### Gaps in the utopia: empirical evidence on Empirical ’s development and regional impacts

While the official Masterplan presents a cohesive sustainability vision, recent research reveals significant tensions between these planning ideals and the projected on-the-ground dynamics. Scholars have shifted from descriptive accounts toward predictive modeling, highlighting how Nusantara’s development generates critical environmental, resource, and social vulnerabilities.

Despite the Forest and Sponge City branding, multi-hazard assessments indicate that the interaction of urban expansion and intensifying climate variables will heighten the risks of flooding, landslides, and heat stress within both Nusantara and its hinterlands.^12^^,^^20^ These physical risks intersect with mounting resource pressures; spatial analyses report declining forest cover and landscape fragmentation in Penajam Paser Utara (PPU) and Kutai Kartanegara (Kukar), specifically along planned infrastructure corridors.^21^ Furthermore, the overlap of development zones with critical habitats jeopardizes the ecosystem services underpinning the government’s visions.^6^^,^^22^ Although suitability modeling suggests that much of the capital is technically fit for residential use,^5^ future population pressures will likely drive expansion into environmentally vulnerable fringes.^23^ Simultaneously, Arman et al.^24^ forecast that demographic growth could trigger a regional rice deficit unless agricultural hinterlands are restructured. These studies suggest that the spatial configuration of new infrastructure will be decisive for maintaining the region’s ecological integrity, hydrological resilience, and food security.

At the regional scale, Nusantara is designed to operate as an economic superhub within a tri-city constellation alongside Samarinda and Balikpapan. Aisyah et al.^25^ and Tarassyta et al.^26^ identified the service quality and comfort of inter-city transport routes—particularly the Samarinda-Balikpapan corridor—as critical determinants of regional accessibility and the subsequent restructuring of the regional economy. This reconfiguration signals a shift from extraction-based activities toward service-intensive sectors, with profound implications for land use along coastal and riverine corridors.^27^ However, evidence of a two-way causality between economic growth and environmental degradation in surrounding regencies suggests that while the superhub drives prosperity, it simultaneously threatens regional environmental quality.^28^ These findings reinforce the argument that Nusantara’s development cannot be evaluated in isolation. Instead, land-use transitions in the surrounding region are structurally coupled to Nusantara’s trajectory, inevitably shaping the cumulative environmental and social outcomes of East Kalimantan.

Beyond biophysical and economic constraints, another strand of literature highlights tensions within the emerging social fabric of the new capital. Wadipalapa et al.^29^ argue that the accelerated development timeline is driven more by symbolic state-building than by technocratic deliberation, potentially embedding long-term institutional weaknesses. Survey-based research with Indigenous communities reveals significant fears of marginalization and a lack of recognition for existing cultural and economic practices in official spatial plans.^30^ These findings suggest that the risks of gentrification and social exclusion directly conflict with its inclusive branding. In this context, the benefits of new infrastructure are likely to concentrate within the administrative core, while the costs of externalities are disproportionately borne by peripheral and Indigenous populations. Consequently, Nusantara’s urbanization is characterized as socially differentiated, producing uneven development outcomes that challenge its sustainable aspirations.

Collectively, the literature on Nusantara’s development yields two critical insights. First, empirical studies reveal that early LUC has already accelerated along strategic corridors and within environmentally sensitive areas, questioning the robustness of existing spatial controls. Second, current predictive models present fragmented, theme-specific scenarios that lack integration into a comprehensive indicator system—one capable of simultaneously capturing physical suitability, environmental limits, and social risks across the expanding agglomeration. While existing research identifies these risks, it has yet to develop a systematic framework to anticipate where these pressures will spatially manifest. This limitation necessitates a methodological shift from single-sector analysis toward an integrative approach that jointly considers the drivers of urban expansion and the socio-ecological boundaries required to contain it. Consequently, a contextualized predictive-preventive framework—incorporating regional ecological thresholds and social vulnerabilities—is essential for projecting Nusantara’s urbanization trajectories and fostering long-term resilience.

## Methods

### Study area: Nusantara and surrounding region

**Figure 2:**
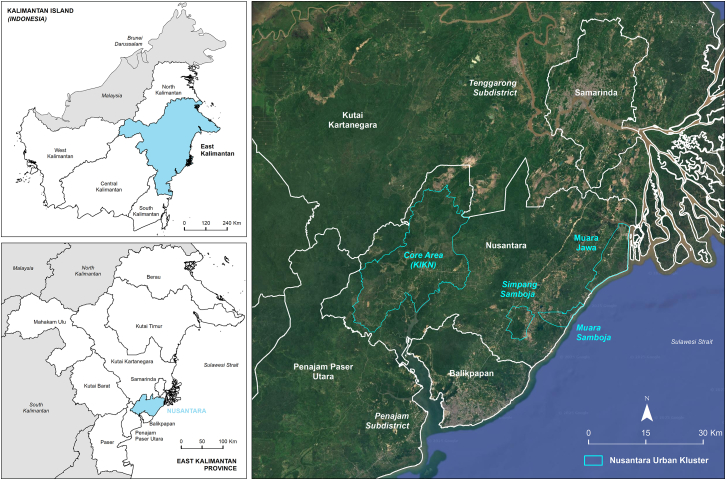
Location map of East Kalimantan Province and Nusantara

The study area encompasses Nusantara, Indonesia’s new capital city in East Kalimantan, and its broader urbanizing region, including PPU and Kukar Regencies, alongside the strategic gateway cities of Balikpapan and Samarinda (Figure 2). Currently, Nusantara’s core governmental zone (KIPP) and wider capital area (KIKN) are characterized by a mosaic of production and protection forests, with existing settlements—such as Sepaku, Samboja, Muara Jawa, and Loa Janan—functioning as ancillary urban clusters. Strategic planning positions this landscape to evolve into a forest-based, water-sensitive, and digitally governed capital that serves as an economic superhub within the Nusantara-Balikpapan-Samarinda corridor. The confluence of intense development pressure and significant remaining forest cover renders Nusantara and its hinterland a critical laboratory for analyzing LUC, spatial interactions, and sustainability trade-offs in NCD.

### Literature analysis: review of existing LUC research

**Figure 3:**
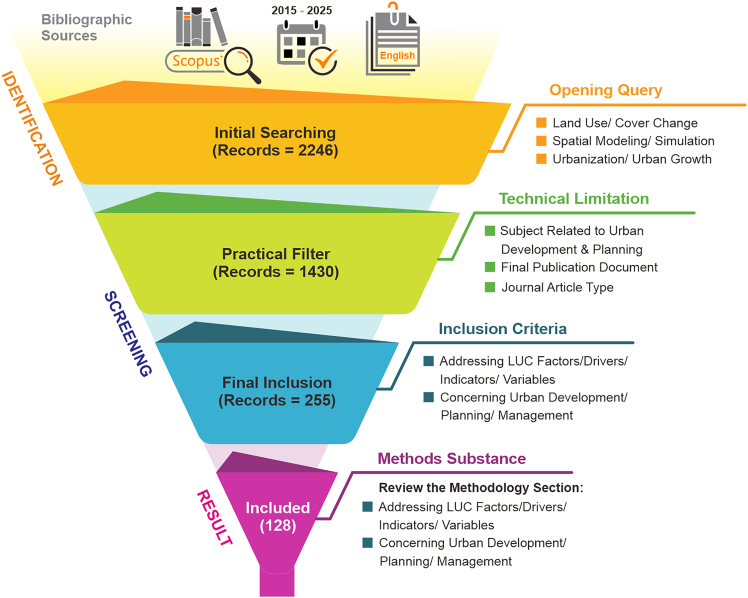
Search and screening workflow for literature review

We conducted a literature analysis to synthesize predictive indicators in current LUC research and their applicability to the Nusantara agglomeration. Following Ghozali and de Vries^31^ methodological approach, we used Scopus as the primary bibliographic source and applied a structured multi-level keyword query searching across titles, abstracts, and author keywords (Table 1). The search was initially restricted to English articles published between 2015 and 2025 and specifically within keywords relevant to land use land cover dynamics, spatial modeling, urbanization, and urban growth. We mitigated potential selection bias and ensured contextual relevance by restricting the analysis to final-version publications indexed within thematic categories pertaining to urban development and spatial planning. Following the implementation of the primary inclusion search, an initial retrieval yielded 255 records, which were subsequently screened for substantive relevance. Inclusion criteria targeted studies that explicitly examined LUC factors, indicators, or drivers associated with urban expansion modeling in a planning and development context. The selected records were then reviewed manually to ensure that the inclusion criteria were applied consistently and described clearly in the methodology section. Articles lacking methodological or thematic alignment were excluded, resulting in 128 articles for subsequent analysis. The complete retrieval and screening workflow is illustrated in Figure 3.

**Table 1:** Final query for searching and screening literature from Scopus database

Final query string
(TITLE-ABS-KEY ((“land use” OR “land cover” OR lucc OR lulc∗ OR luc OR spatio∗) W/4 (change∗ OR dynamic∗ OR transition∗ OR transformation∗)) AND TITLE-ABS-KEY ((land OR lucc OR luc) W/3 (future OR (predict∗) OR (model∗) OR (project∗) OR (simulat∗))) AND TITLE-ABS-KEY ((“urban growth” OR “urban expansion” OR “city growth” OR (urbaniz∗) OR (urbanis∗) OR “urban transformation”)) AND TITLE-ABS-KEY ((factor OR variable OR driver OR contributor OR “driving force” OR indicator OR parameter)) AND TITLE-ABS-KEY ((urban OR city OR town OR regional) W/3 (development OR planning OR management))) AND PUBYEAR >2014 AND PUBYEAR <2026 AND (LIMIT-TO (DOCTYPE, “ar”)) AND (LIMIT-TO (PUBSTAGE, “final”)) AND (LIMIT-TO (LANGUAGE, “English”)) AND (LIMIT-TO (SUBJAREA, “ENVI”) OR LIMIT-TO (SUBJAREA, “SOCI”) OR LIMIT-TO (SUBJAREA, “AGRI”) OR LIMIT-TO (SUBJAREA, “EART”) OR LIMIT-TO (SUBJAREA, “ENGI”) OR LIMIT-TO (SUBJAREA, “DECI”) OR LIMIT-TO (SUBJAREA, “ECON”) OR EXCLUDE (SUBJAREA, “ENER”) OR EXCLUDE (SUBJAREA, “COMP”) OR EXCLUDE (SUBJAREA, “MEDI”) OR EXCLUDE (SUBJAREA, “BUSI”) OR EXCLUDE (SUBJAREA, “BIOC”) OR EXCLUDE (SUBJAREA, “PHYS”) OR EXCLUDE (SUBJAREA, “NEUR”) OR EXCLUDE (SUBJAREA, “MATH”) OR EXCLUDE (SUBJAREA, “MATE”) OR EXCLUDE (SUBJAREA, “IMMU”) OR EXCLUDE (SUBJAREA, “CHEM”) OR EXCLUDE (SUBJAREA, “CENG”)) AND (LIMIT-TO (SRCTYPE, “j”))

From each article, we extracted, categorized, and tabulated the reported LUC indicators as fundamental keywords in Microsoft Excel. To address the diverse terminology across the literature, we standardized the terms to ensure conceptual comparability. VOS viewer 1.6.20 was then employed to conduct a bibliometric analysis based on keyword co-occurrence, generating a density map of emerging indicator-related keywords.^32^ A frequency threshold of more than two occurrences was applied to prioritize indicators commonly utilized in LUC research. This density mapping facilitated the visualization and exploration of research hotspots and thematic clusters related to urban expansion indicators.^33^ This process provided a consolidated pool of generic LUC indicators for further analysis and evaluation. Due to the limitations of VOS viewer in producing publication-quality visualizations, CorelDRAW version 24 was used to enhance image resolution without altering the underlying results. This software was also used to develop the framework figures presented in the final synthesis.

### Translating literature-based LUC indicators to the Nusantara agglomeration context

We applied a contextualization and integration process to assess the relevance of LUC indicators to the Nusantara and East Kalimantan context through a synthesis of three sources of input. First, the global LUC literature provided the generic indicator pool derived in the literature analysis subsection. Second, local empirical studies on Nusantara and East Kalimantan were used to assess empirical support and contextual relevance. Unlike the global literature review, which relied on Scopus, the local literature corpus on spatial, social, and economic dynamics was assembled through targeted web searches using the terms “IKN Nusantara,” “Indonesia New Capital,” and “Indonesia Capital Relocation.” Only credible journal articles and conference proceedings were retained. Third, the Nusantara Development Masterplan was used as the primary planning document to interpret the strategic priorities and spatial intentions of the Forest-Sponge-Smart development vision.

Initially, indicators were categorized as either predictive or preventive based on their primary function in LUC assessment. Predictive indicators were defined as explanatory variables for land-conversion suitability and urban growth trends, capturing internal drivers or inhibitors through direct, proximity-based, or derived metrics. Preventive indicators were defined as analytical variables representing specific urban issues and interactions that regulate, moderate, or redirect expansion probabilities.^31^

Next, predictive indicators across the five established categories—geophysical foundation (GP), climate interaction (CC), economic and demography (ED), urban functionality (UF), and institutional policy (IP)—were evaluated against local empirical studies to verify their relevance and data applicability. Each indicator was assessed according to its documented influence on regional spatial dynamics and its alignment with the current development phase of the Nusantara agglomeration. Indicators meeting these criteria were retained, while overlapping indicators were merged and those lacking local relevance or measurable proxies were discarded.

We then considered candidate preventive indicators that capture urban challenges exceeding internal dynamics—specifically environmental sustainability and regional inequality. Each candidate was screened for conceptual alignment with the masterplan-defined strategy, relevance to regional capacity and uneven development, and distinct analytical value relative to the retained predictive indicators in the Nusantara agglomeration. Only indicators satisfying all three criteria were included in the preventive set.

Finally, we synthesized these contextualized predictive and preventive indicators into an integrated LUC indicator framework tailored to the Nusantara agglomeration. In this framework, predictive indicators represent land-conversion suitability and urban growth propensity, whereas preventive indicators regulate or moderate conversion probabilities in relation to regional sustainability and uneven development. The framework is therefore proposed conceptually as an integrative synthesis of these evidence streams, rather than as a direct empirical finding or a restatement of planning documents. It provides the methodological link between the international LUC literature and Nusantara’s development vision, supporting the interpretation of future urbanization trajectories and informing proactive strategies for sustainable land-use management in the region.

## Results

### Existing LUC research and prevalent LUC indicators

**Figure 4:**
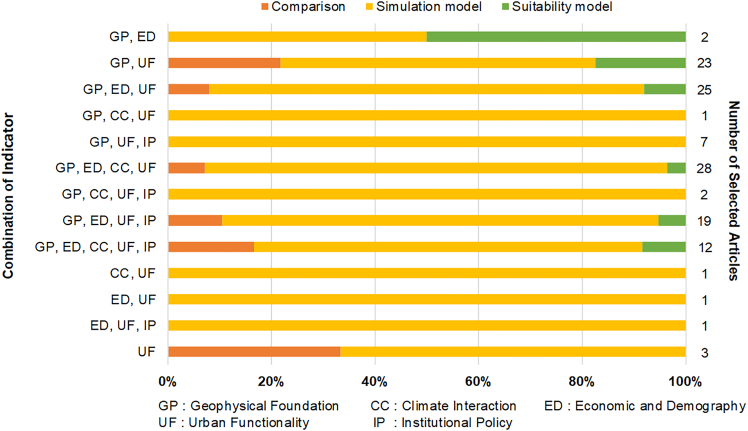
Methodological diversity in LUC research and the combination of indicators used

Over the past decade, the number of LUC studies has increased markedly. Illustrated in Figure 4, scenario-based simulations dominate the field, employing diverse LUC indicators to forecast future land-use patterns under varying socio-ecological manipulations. For instance, Fu et al.^34^ integrate remote sensing, socioeconomic, and meteorological datasets to simulate urban expansion and its subsequent impacts on carbon storage. In contrast, comparative modeling remains relatively sparse, typically focusing on cross-validating simulation tools or refining factor-weighting techniques to enhance predictive accuracy—exemplified by the Cycle-Gated Recurrent Unit framework.^35^ Validation-focused research represents a smaller fraction of the literature, prioritizing the empirical reliability of specific drivers and constraints over long-term forecasting. Collectively, this indicates a research landscape that is technically advanced yet fragmented across specific modeling paradigms.

**Figure 5:**
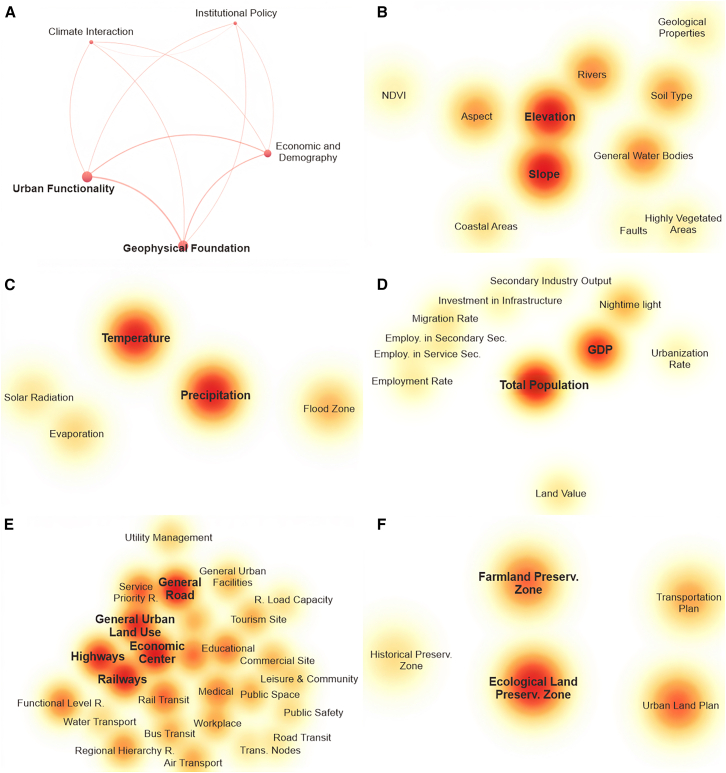
Network visualization and density maps of co-keyword occurrences across the five predictive indicator categories (A) Connection visualization between Indicator categories across collected literature. The figure highlights the relative concentration and overlaps of indicators within (B) GP, (C) CC, (D) ED, (E) UF, and (F) IP, showing how global LUC studies emphasize different dimensions of urban growth and land transformation. Node sizes can only be compared within the same category.

Despite their methodological diversity, these models reveal the prevalent LUC indicators in various global case studies. Within the reviewed articles, we observe distinct trends in indicator group combinations. For instance, Rodrigues et al.^36^ utilized only GP and UF, whereas Zu et al.^35^ employed the complete set of indicator groups. In Figure 4, GP indicators are the most frequent and demonstrate a strong association with UF. This pair exhibits significantly higher network strength than other combinations (Figure 5A), reflecting the dual dynamic where GP indicators regulate expansion while UF indicators stimulate it. Compositions that incorporate ED drivers have also gained traction, capturing the links between land conversion and socioeconomic shifts. In contrast, combinations involving CC and IP remain under-represented, as indicated by their limited occurrences (Figure 4) and the sparse connections (Figure 5A). This deficit suggests that mainstream LUC modeling continues to overlook the critical roles of climate variability and institutional frameworks in shaping land dynamics.

Figures 5B–5F present the density visualization of indicators across five categories: GP, CC, ED, UF, and IP. Each point represents the LUC indicator and density gradients ranging from low (white-yellow) to high (orange-red), derived from keyword frequency and co-occurrence. These visualizations highlight both overlaps and distinctions in how global studies utilize indicators within these clusters. While physical, social, and economic drivers remain dominant in LUC studies, preventive dimensions such as climate resilience and policy enforcement are emerging as vital complements, which are gaining prominence as robust and context-specific metrics for sustainable land management.

The GP category (Figure 5B) consists of landscape characters, with elevation and slope exhibiting the strongest network strength, reinforcing their status as fundamental natural constraints in LUC modeling. Similarly, the CC category (Figure 5C) reveals the notable co-occurrence between rainfall, temperature, and climate hazards, underscoring a shift toward risk-sensitive development. The ED category (Figure 5D) forms a dense cluster around population growth, GDP, and economic activity—the latter often proxied by Nighttime Light (NTL) intensity. In the UF category (Figure 5E), keywords regarding urban centrality, amenities, and accessibility are prominent, highlighting the role of spatial proximity in shaping growth. While macro-level studies typically employ generalized metrics such as road density, micro-level analyses adopt finer indicators such as transportation hierarchy, facility typologies, and land use variation. Finally, although less prevalent, the IP category (Figure 5F) exhibits the moderating impact of zoning and ecological protection on urban transformation.

### Contextualization of predictive indicators in the Nusantara agglomeration

#### Geophysical foundation

The reviewed literature suggests that GP indicators capture the natural characteristics of landscapes and landforms, which guide land-use decisions. Indicators in this category consistently delineate the physical limits of urban expansion. Elevation and slope are decisive factors in determining development suitability, as they define feasible construction zones while supporting ecological preservation.^37^ Similarly, soil type and geological properties dictate land productivity and stability, influencing the suitability of areas for agriculture, settlements, and heavy infrastructure.^38^^,^^39^ Hydrological and vegetation-related factors—including proximity to water bodies, wetlands, and forests—determine water resource availability and simultaneously regulate flood susceptibility.^40^ Additional attributes, such as slope orientation and vegetation indices, further influence microclimates and biodiversity conservation.^41^ These indicators clarify how physical geography regulates LUC by establishing the foundational suitability of local conditions for urban development.

In East Kalimantan, the role of physical geography in shaping urban growth is particularly evident. The province’s diverse topography—ranging from coastal plains and inland hills to extensive river estuaries^42^ —historically guided the spatial structures of Balikpapan on the southern coast and Samarinda along the Mahakam River. The relocation of the capital to Nusantara reflects the perceived advantages of this landscape, specifically its stable biophysical conditions and relatively low disaster risk.^43^ Although short-active faults exist in Paser and Kutai Timur, they remain distant from Nusantara and exhibit low seismic potential.^44^ Nonetheless, the extensive protected forests encircling the capital act as both ecological constraints and vital assets for the Forest City vision.^6^ Consistent with global scholarship, this context underscores the decisive role of nine specific GP indicators in shaping Nusantara’s land-use trajectory, as detailed in Table S2.

#### Climate interaction

The integration of CC indicators in LUC modeling remains limited due to their indirect mechanism on urban development. Nonetheless, five indicators are commonly recognized in the literature: temperature, precipitation, solar radiation, evaporation, and flood zones. Among these, temperature and precipitation serve as critical determinants, regulating water availability and soil moisture, which are essential for sustaining settlements.^45^ Urbanization, in turn, often amplifies the Urban Heat Island (UHI) effects^46^ and exacerbates extreme rainfall.^47^ Although less frequently emphasized, solar radiation and evaporation function as proxies for hydrological cycles, reflecting energy-moisture exchanges that dictate drought risk.^48^ Furthermore, flood zone proximity emerges as a decisive factor, directly coupling climate hazards with spatial outcomes. High flood exposure frequently restricts development in high-risk zones while necessitating the adoption of climate-resilient infrastructure.^49^ Collectively, these indicators demonstrate how climate processes act as both a constraint on and a driver of land-use patterns.

In the case of Nusantara, previous studies confirm a strong correlation between temperature and LUC. Current projections indicate that extensive development and forest encroachment will exacerbate UHI effects across the region.^10^^,^^50^ Furthermore, rainfall patterns in Nusantara and adjacent regions indicate a notable upsurge in coastal zones and a decline in inland areas, heightening the risk of urban clean water shortages and hydrometeorological disasters.^12^ These findings emphasize the necessity of mitigation strategies aimed at climate-resilient urban planning. Although the region is relatively shielded from major geological hazards, it remains highly susceptible to climatic risks, including biodiversity fragmentation, floods, and landslides.^6^^,^^20^ These challenges underscore the importance of integrating CC indicators into LUC assessments, as detailed in Table S2. Ultimately, these indicators provide the analytical capacity to evaluate how Nusantara’s land functions under varying degrees of climate adaptability.

#### Economic and demography

Indicators within the ED category are primarily dominated by population dynamics and financial outcomes (Figure 5D). Population density generates demographic pressures that heighten demand for housing and services, accelerating the conversion of natural land into built-up areas.^51^ Migration further shapes urbanization by redistributing populations and catalyzing new settlement clusters in peri-urban zones.^45^ As infrastructure concentrates in the urban core, rising land prices inevitably push development toward more affordable suburban regions.^37^ NTL data effectively captures these spatial dynamics, utilizing artificial light intensity as a proxy for economic activity and urban footprint.^52^ Therefore, NTL has emerged as a reliable indicator for assessing urban-rural trade-offs and socioeconomic distribution over time.^45^ Furthermore, macroeconomic structures—such as GDP, employment rates, and industrial output—clarify how financial growth translates into land transformation. GDP stimulates expansion through greater capital inflows and economic activities,^53^ while employment shifts from resource-based to service sectors reinforce the transition of land from natural to urban uses.^54^ Finally, infrastructure investment and industrial output demonstrate how capital expenditure directly drives land consumption for new facilities and residential zones.^55^

In Nusantara, empirical evidence confirms that development pressure and economic output are already reshaping regional dynamics. The relocation of civil servants is projected to intensify demographic shifts, significantly increasing land demand for housing and public facilities across the surrounding municipalities.^4^ Beyond the primary migration driven by capital construction, Virtriana et al.^23^ demonstrate that existing high-density areas are already experiencing intensified demand for built-up land, leading to development encroachment into regions with lower environmental suitability. Consequently, population density and migration patterns serve as primary indicators of regional redistribution, concentrating land conversion pressures within each municipality of the agglomeration corridor.

Concurrently, East Kalimantan has undergone a structural pivot from an agricultural to a manufacturing-based economy. While the mining sector’s relative financial contribution has fluctuated, it remains a primary catalyst for growth, leveraging the region’s abundant natural resources.^56^ Capital relocation has further boosted the service and manufacturing sectors, accelerating a transition from resource-intensive industries toward a more diversified economy and expanding employment in secondary and tertiary industries.^57^ Despite recording the second-highest Regional GDP (GRDP) per capita in Indonesia after Jakarta,^27^ significant disparities persist in East Kalimantan between urban centers and rural regencies due to uneven fiscal capacities and infrastructure investment.^58^^,^^59^ Moreover, targeted investment in Nusantara’s urban infrastructure is projected to inflate land values and catalyze real estate speculation in the hinterland.^60^ GRDP and NTL, therefore, serve as vital metrics to bridge macroeconomic performance with localized land dynamics. As shown in Table S3, ten ED indicators collectively define the demographic-economic nexus that will drive future LUC trajectories in the Nusantara agglomeration.

#### Urban functionality

With twenty-seven identified indicators, the UF category is the most extensively utilized in LUC modeling (Figure 5E). These indicators are categorized into three interrelated dimensions: spatial centrality, transportation mobility, and urban amenities, which collectively define the spatial attractiveness of a region. Proximity to economic hubs—including business districts (CBDs), government zones, and administrative cores—serves as a primary predictor of LUC due to the high concentration of infrastructure and accessibility.^61^ Furthermore, proximity to existing built-up or urbanized areas reflects the outward diffusion of urban footprints into the hinterland.^62^ Specific land uses, such as industrial and residential zones, act as catalysts for adjacent commercial development,^38^ while proximity to resource-based areas (e.g., agriculture and mining) dictates land intensity in resource-dependent economies.^63^^,^^64^ Beyond economic hubs, the presence of urban amenities further enhances spatial attractiveness. While essential public services—including education and healthcare—delineate strategic development zones,^65^ commercial amenities such as retail and hospitality increase local land value and investment.^66^ Additionally, public safety infrastructure (police and military offices) and public spaces (parks and tourist attractions) improve the perceived quality of life, fostering further settlement expansion.^67^

Transportation accessibility emerges as another decisive factor in enhancing land attractiveness for development. Well-integrated road networks, railway lines, and transit hubs minimize travel costs, extend metropolitan connectivity, and stimulate transit-oriented expansion.^9^ Strategic nodes—such as terminals, ports, and airports—further intensify economic activity through efficient multi-modal interchanges.^61^ High-capacity infrastructure, including highways and expressways, restructures regional connections by directly promoting axial development and large-scale urbanization.^9^ Recent scholarship also emphasizes the role of road classification, distinguishing between hierarchies that shape distinct traffic flows and land-use integration. For example, Liu et al.^45^ examined how regional networks—ranging from national to district-level roads—differentially influence LUC. Similarly, diverse rail systems, including high-speed rail and subways, offer varying degrees of spatial accessibility, thereby dictating the intensity of urban concentration.^40^

The relationship between UF indicators identified in global literature is highly relevant to the Nusantara context. Development in East Kalimantan across small and medium-sized cities is predominantly driven by mining, manufacturing, and trade.^4^ Consequently, proximity to these hubs catalyzes land transformation, while persistent disparities in infrastructure and accessibility create regional imbalances in centrality and livability.^68^ These facilities—particularly in education, health, commerce, public spaces, transportation, and utilities—are critical elements of urban life that actively promote rural-to-urban transitions. Furthermore, road-based networks remain the primary driver of mobility-led growth; specifically, the Balikpapan-Samarinda toll road has restructured the regional economy,^69^ and benefiting intercity connectivity in adjacent areas.^26^ Among the Indonesian road hierarchy, the functional classification of roads—ranging from arterial to local—exerts a unique influence on urban development by dictating traffic efficiency and the intensity of surrounding economic activity.^70^

These indicators collectively establish a framework for assessing spatial attractiveness across the agglomeration. Summarized in Table S3, seventeen indicators are directly applicable to the Nusantara context. Others were excluded due to categorical incompatibility with the current socio-spatial profile. For instance, while certain specialized urban facilities improve livability in megacities, workplace-specific and leisure amenities were excluded. The cities within the Nusantara agglomeration remain predominantly small to medium in scale, meaning such amenities are not yet among the community’s primary needs.

#### Institutional policy

The density mapping of IP-related keywords (Figure 5F) identifies six primary indicators: urban development zones, transportation plans, historical preservation zones, ecological preservation zones, and agricultural protection zones. These factors illustrate the dual nature of institutional frameworks, which function as either developmental enablers or restrictive systems. Urban development zones, such as special economic zones and industrial estates, serve as formal incentives to channel expansion and cultivate emerging economic centers.^71^ Strategic transportation plans, including toll roads and new arterial networks, stimulate regional economies and fundamentally restructure land-use patterns.^53^ Conversely, restrictive regulations establish exclusion zones that halt urban sprawl in vulnerable or high-value landscapes. Forests, wetlands, biodiversity hotspots, prime farmlands, and heritage sites necessitate these policy interventions to enforce conservation priorities.^72^ These indicators illustrate how governance frameworks dictate spatial outcomes, providing the basis for assessing regulatory mechanisms within the Nusantara context.

While large-scale infrastructure and expanding road networks have catalyzed new economic hubs, scholars have increasingly highlighted the resulting socio-ecological trade-offs.^4^^,^^30^ The project’s spatial footprint risks accelerating biodiversity loss and threatening endemic fauna.^6^ This ecological strain is compounded by urban encroachment and tourism expansion, which jeopardize indigenous Dayak cultures and the heritage of the Kutai kingdom.^73^ Furthermore, the region faces a burgeoning food security challenge, as current agricultural capacity remains insufficient to sustain the projected population influx.^74^

Beyond development impacts, scholarship has increasingly documented the implementation of these restrictive measures. High-priority protected forests within and around Nusantara require stringent, long-term conservation to maintain the Forest City integrity.^5^ Similarly, food security remains a primary governance concern, as evidenced by national agricultural protection policies designed to safeguard prime farmlands from urban encroachment.^75^ Although often perceived as rural issues, these protective mandates are now formally integrated into the spatial plans of Samarinda, Balikpapan, PPU, and Kukar. Furthermore, cultural heritage protections are embedded across municipal frameworks to preserve regional identity amidst rapid transformation.^76^^,^^77^ Overall, IP indicators serve as the regulatory pivot for the region: While development zones attract capital and opportunity, ecological and cultural policies function as the essential preventive mechanism for sustainable growth.

### Synthesis of contextualized predictive indicators

The contextualization established in the contextualization of predictive indicators subsection demonstrates that the predictive indicators relevant for the study region constitute a coherent system of physical constraints, spatial attractiveness, and institutional controls. Table 2 summarizes these retained indicators, delineating their specific roles in the urbanization process and their direct applicability to LUC modeling within the Nusantara context.

**Table 2:** Summary of the full predictive indicator set contextualized for the Nusantara agglomeration

Group	Hierarchy level	Retained LUC factors with occurrence counts^a^	Role in shaping Nusantara urbanization	Role in LUC modeling
UF	direct core predictors	• Proximity to economic and administrative centres (57) • Existing built-up areas (45) • Industrial zones (21) • Mining areas (9) • Education facilities (20) • Medical facilities (18) • Public space facilities (10) • Tourism sites (10) • Commercial sites (9) • Utilities infrastructure (5) • Public safety facilities (4) • Toll-roads (45) • Bus terminals (10) • Airports (10) • Ports (4) • Road transit, e.g., toll gates (3) • Accessibility to Functional-class roads (20)	Represent spatial magnetism, measuring how proximity to transport networks, economic hubs, and amenities channels and concentrates urban expansion.	Accessibility and attraction-based drivers
ED	Indirect core predictors	• Population density (83) • Regional GDP (52) • NTL (13) • Migration rate (4) • Land value (4) • Employment rates (3) • Employment in service sector (3) • Employment in secondary sector (2) • Industrial output (2)	Indicate where land demand and structural change are strongest, reflecting population growth, migration, and shifts toward manufacturing and service sectors.	Demand-related transition determinants
GP	Foundational constraint predictors	• Elevation (105) • Slope (113) • Proximity to rivers/coast (64) • Aspect (27) • Soil type (25) • Geological properties (4) • Forest/highly vegetated areas (4) • NDVI (3)	Delineates the biophysical foundation and terrain constraints, distinguishing developable land from ecologically sensitive, steep, or geologically unstable landscapes.	Suitability variables/physical constraints
CC	Supporting constraint predictors	• Temperature (36) • Precipitation (33) • Evaporation (3) • Solar radiation (2) • Proximity to flood-prone zones (6)	Identifies climatic thresholds and hydrometeorological hazards, highlighting areas where environmental risks necessitate either development restrictions or resilience-driven design.	Risk-sensitive suitability variables/climate constraints
IP	Regulatory modifiers	• Urban development zones, e.g., new urban areas and special economic zones (13) • Transportation plans (6) • Ecological preservation zones (28) • Agricultural protection zones (12) • Historical and cultural heritage zones (2)	Encode planning decisions as the regulatory pivot, balancing growth-enabling development zones with preventive ecological, agricultural, and cultural safeguards.	Policy-related suitability variables/exclusion constraints

a Numbers in parentheses indicate the occurrence of each indicator in the reviewed studies. These values show empirical prevalence in the literature and are intended to support initial prioritization in future applications, not to imply a universal ranking of analytical importance.

Beyond their empirical utility, these indicator groups fulfill distinct methodological functions and form a practical hierarchy within the LUC modeling framework. UF indicators act as direct core predictors representing spatial magnetism and proximity-based attraction, which directly dictate the location of urban expansion. Meanwhile, ED indicators function as indirect core predictors, capturing the market pressures and demographic demands that intensify land conversion. These indicators therefore represent the central variables for modeling future urbanization in the Nusantara agglomeration by integrating spatial attraction with demand-driven transition likelihoods.

In contrast, GP and CC indicators primarily function as constraint predictors. GP indicators serve as foundational constraints, defining the biophysical conditions that determine the basic land suitability for development. CC indicators refine this suitability by incorporating climate sensitivity and hydrometeorological risk. While essential, these categories function to condition or differentiate where growth can occur rather than generating growth pressure itself. Finally, IP indicators operate as regulatory modifiers, whose analytical influence depends on the enforceability of zoning, infrastructure plans, development incentives, and exclusion areas. Consequently, these can be operationalized as policy levers to simulate contrasting development scenarios.

These groups encapsulate the essential dimensions of suitability, demand, accessibility, and governance required to project urbanization dynamics in the Nusantara agglomeration. This hierarchy establishes that the predictive indicator set should not be treated as an undifferentiated list, but as a structured variable system. Within this system, core predictors guide baseline transition probabilities, constraint predictors delimit feasible or risk-sensitive development zones, and regulatory modifiers calibrate outcomes based on specific planning and policy scenarios.

## Discussion

### How future urbanization unfolds in the Nusantara agglomeration: a conceptual framework

#### Urbanization from individual entity force

**Figure 6:**
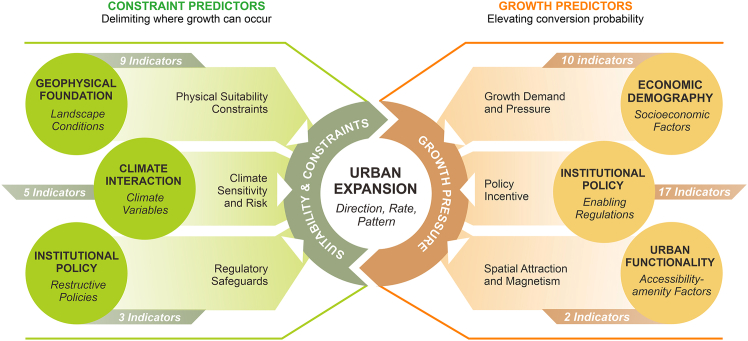
Predictive indicator framework for future urbanization in the Nusantara agglomeration The Category circles define the indicators' contents, while the extending arms delineate their specific functions within the LUC modeling framework. The central ring represents the fundamental indicators' interaction that reinforces internal urban expansion. The left side illustrates how constraint predictors delimit potential growth areas by defining land-conversion suitability. The right side demonstrates how growth predictors elevate the probability of land conversion within the urban system.

As detailed in the synthesis of contextualized predictive indicators subsection, the selected indicators demonstrate strong theoretical consistency with the Nusantara agglomeration, effectively characterizing the internal conditions that shape future urbanization. The combination of physical landscape characteristics, climate conditions, socioeconomic factors, UF, and institutional regulations determines where urban land emerges, how rapidly it expands, and which spatial patterns are likely to manifest.^31^ These indicators function as internal forces that position each municipality as a growth node, derived from the dynamic interplay between expansionary drivers and spatial constraints. Following the hierarchy established in Table 2, UF and ED indicators serve as core growth predictors, GP and CC indicators as constraint predictors, and IP indicators as regulatory modifiers. Figure 6 visualizes this hierarchy, illustrating the interacting mechanisms between these spatial constraints and growth drivers.

Predictive indicators translate the distinct internal dynamics of Nusantara and its surrounding municipalities into measurable components. ED (10 indicators) represents a socio-demographic nexus and development pressure that generate demand for land conversion and infrastructure. Simultaneously, UF (17 indicators) measures centrality, accessibility, and amenity distribution that support economic concentration and enhance urban livability. The growth-driving nature of these ED and UF indicators serves as a primary metric for modeling the pattern and rate of urban expansion (Figure 6). Consistent with the occurrence patterns reported in Table 2, population growth, economic activity, and accessibility—key elements of ED and UF—remain core variables in empirical LUC models. This development trajectory reflects a common mechanism whereby urban expansion tends to follow concentrations of jobs, services, and infrastructure. Complementing these forces, incentive-based policy, such as new development zones and strategic infrastructure projects, functions as institutional enablers. By attracting investment and accommodating housing demand, these systems catalyze the emergence of new economic centers, actively stimulating further urban growth.

As a national strategic NCD initiative, the Nusantara Development Masterplan positions the construction within a defined boundary as a greenfield development, which becomes a powerful internal attractor. The plan envisions a large-scale conversion of green landscapes in PPU and Kukar into a comprehensive urban core comprising government, commercial, and residential zones.^4^ The massive infrastructure development corroborates findings in the UF subsection, confirming that new economic and transport hubs strengthen spatial attractiveness. Concurrently, the relocation of civil servants triggers an influx of skilled labor, raising demand for housing and services throughout the region.^78^ These mechanisms shape internal urbanization within the capital’s planned boundaries.

The phased implementation of Nusantara also reconfigures the regional socioeconomic landscape. While Balikpapan and Samarinda retain established internal systems, new growth hubs rapidly emerge in adjacent regions through the transformation of nearby villages into peri-urban zones.^79^ Nusantara development accelerates early regional development, which is already concentrated along strategic transport corridors and nascent service hubs across municipalities.^5^ Further empirical evidence underscores this impact, documenting rising land prices,^60^ and a structural shift toward manufacturing and service-oriented activities in adjacent regions.^57^ These dynamics illustrate how Nusantara intensifies existing amenities in surrounding areas, thereby reinforcing spatial growth poles at the municipal scale.

However, urban expansion is not governed solely by growth forces. It is equally constrained by environmental suitability and climate adaptability, such as geological stability and hydrological risks. GP (9 indicators) and CC (5 indicators) delineate the technical feasibility of new construction, dictating where development must be avoided. Complementing these physical limits, restrictive institutional policies, such as ecological preservation and agricultural protection zones, enforce boundaries on urban sustainability. Although each municipality possesses distinct environmental limitations, development guidelines within Nusantara exert a dominant influence across the agglomeration. Within the capital, spatial guidelines derived from the Forest City and Sponge City visions actively curate development, confining growth to designated zones. Yet, unplanned expansion beyond these boundaries, particularly near KIPP, risks undermining the capital’s administrative function. Consequently, development pressure spills over into adjacent border regions, particularly along major corridors with higher intensities.^79^

While the predictive indicators framework implies a theoretical equilibrium between driving and inhibiting mechanisms, the application of internal urbanization is often constrained by administrative jurisdictions. This limitation means that *trans*-municipal interactions elude capture when relying solely on nodal drivers and local constraints. Ultimately, without a robust restrictive system (GP, CC, IP-Restrictive) to counterbalance powerful driving forces (ED, UF, IP-Enabler), internal urbanization is projected to exceed its planned boundaries.

### Urbanization from regional interactions

Regional interactions function not merely as external factors but as spatial conduits that translate node-level impulses into cross-boundary expansion. In Nusantara, these dynamics are driven by functional distribution, proximity, and infrastructure, creating an emerging agglomeration where complementary districts anchor distinct regional roles beyond the KIPP.^58^^,^^59^^,^^79^ Specifically, the decentralization of activity centers propels the growth of specialized hubs and local attractions, such as the food industry in Kuala Samboja, Agriculture and Fisheries in Simpang Samboja, and Muara Jawa as a rural settlement consolidation.^8^ At the macro scale, this structural shift is reinforced by highway and railway expansions that enhance connectivity with adjacent municipalities, facilitating the demographic mobility essential to urban agglomeration.^80^ While the resulting Nusantara-Balikpapan-Samarinda superhub fosters economic integration and built-up growth, it simultaneously precipitates critical externalities—including environmental degradation,^60^ and rural-urban disparities^59^—that necessitate deliberate policy intervention.

To mitigate these challenges, the Nusantara Masterplan operationalizes Forest-Sponge-Smart City principles, utilizing conservation to curb sprawl and the Zero Delta Q principle to enhance hydrological resilience through intelligent governance. However, current planning instruments remain tethered to conventional regulatory approaches that prioritize internal capital development over regional integration. When interpreted through regulatory-based indicators, the Masterplan demonstrates robust internal governance but fails to account for development externalities along regional corridors. This insular perspective treats cities as self-contained units, ultimately hindering spatial coordination and obscuring critical cross-boundary spillovers.^81^^,^^82^

Therefore, future urbanization assessments must harmonize Nusantara’s design strategies with measurable LUC indicators at the agglomeration scale to capture these regional dynamics. Although underutilized, preventive indicators are emerging as critical adaptive measures capable of quantifying regional interactions and capacities.^31^ In LUC simulations, these indicators condition rather than stimulate growth, acting as resistance layers or weighting modifiers to lower transition probabilities in ecologically fragile or socio-spatially unequal areas. Unlike predictive indicators, which identify where development pressure intensifies, preventive measures dictate where such pressure should be curtailed or redirected to avoid unsustainable transformation. Rather than functioning as fixed rule-based masks in conventional policy, they provide spatially explicit representations of environmental limits and uneven development. Recent frameworks have operationalized this through metrics such as Ecological Resistance Quality and Land-Use Conflict Potential,^83^ while others have enhanced accuracy by integrating Spatial Development Heterogeneity^84^ and Urban Gravity.^66^ These indicators complement predictive drivers and offer a responsive simulation of cross-boundary externalities.^38^

By translating Nusantara’s development principles into measurable safeguards, specific preventive indicators can be incorporated into LUC models to capture emerging urban challenges. Table 3 summarizes the contextualization of preventive indicators, including their strategic alignment and possible role in future LUC modeling. While each indicator possesses a distinct strategic alignment, its applicability necessitates context-specific adaptation. For instance, spatial intensification metrics—such as Tourism and Compact Development Assessment—are primarily effective in mature metropolitan areas where infill is central to sprawl mitigation.^38^^,^^90^ Conversely, such metrics remain less applicable to Nusantara, which is currently characterized by dispersed small-to-medium centers, developing infrastructure, and nascent tourism functions.

**Table 3:** Summary of the full preventive indicator set contextualized for the Nusantara agglomeration

Indicators	Sub-indicators	Reference	Nusantara vision alignment	Contextual relevance	Role in LUC modeling
**Connecting issue: environmental sustainability**					

Ecological resistance quality (ERQ)^a^	• Soil erosion sensitivity • Biodiversity service • Water conservation service • Soil and water conservation service • Vegetation coverage • Forest density • Water yield • Water network density • Water intersection density • Geological and flood risks	Gao et al.^81^; Wang et al.^83^; Wang et al.^85^	Forest City, Sponge City	the development of Nusantara carries inherent ecological risks that necessitate the proactive Integration of environmental constraints into land management.^4^^,^^6^ These indicators support the ecological protection vision by identifying critical zones for preservation and reorganization, thereby minimizing land-use conflicts and ensuring growth does not exceed the region’s ecological carrying capacity.	Resistance layer
Land use conflict potential (LUCP)^a^	• Spatial complexity • Spatial fragility • Spatial stability	Wang et al.^83^	Forest City, Sponge City	Integrated into the ERQ indicator to evaluate spatial fragility and land-use compatibility. This variable identifies areas experiencing high environmental stress or competing land functions for stabilizing the socio-ecological landscape.	Resistance layer/threshold constraint
Urban development land-use suitability (UDLS)^a^	Indicators in the GP and CC categories	Wang et al.^83^; Luan et al.^86^; Valencia et al.^87^	Forest City, Sponge City	Considered under the ERQ indicator to align development intensity with ecological thresholds. This ensures that urban exploration areas are delineated based on their inherent suitability.	Suitability constraint/weighting modifier
Sponge City Assessment (SCA)	• Environmental risk index • Hazard vulnerability • Rain-flood resilience index	Yang et al.^88^; Luo et al.^89^	Sponge City	Recognizing East Kalimantan’s high vulnerability to flooding, this indicator translates the Sponge City vision into a measure of regional land adaptability. It links land-use dynamics with hydrological resilience to mitigate rainfall-flood risks in rapidly urbanizing catchments.	Constraint surface/weighting modifier
Tourism development assessment (TDA)	• Proximity to tourist regions • Tourism capacity	Yujie et al.^90^	Not aligned	Metrics focused on urban densification currently hold limited applicability. As Nusantara is a greenfield development in its initial construction phase, spatial intensification is not yet a primary driver; the model prioritizes expansion patterns over the maturation of nascent urban cores.	Not aligned

**Connecting issue: regional inequalities**					

Spatial development heterogeneity (SDH)	Indicators in the UF category	Qian et al.^9^; Jamali et al.^62^; Huang et al.^84^	Smart City, Sponge City	captures the pronounced economic and urbanization disparities within the region, ranging from the rapid growth in Balikpapan and Samarinda to the slower development in PPU and Kukar.^59^ This indicator monitors whether concentrating smart and sponge infrastructure in the core inadvertently amplifies regional imbalances or creates enclave growth patterns.	Weighting modifier/scenario penalty
Urban gravitational interaction (UGI)	• Infrastructure density • People-traffic-information flow intensity • Local accessibility zonal accessibility	Xu et al.^66^; Gao et al.^81^; Liu et al.^91^	Smart City	Reflects the powerful spatial linkages driven by the population, accessibility, and economic capacity of the East Kalimantan corridor.^11^ As Nusantara’s infrastructure matures, this indicator tracks the intensification of inter-urban connectivity, modeling how spatial interdependence accelerates land-use transitions across municipalities.	Weighting modifier/interaction-based modifier
Urban compactness (UCD)	• Urban land density • Mixed-use development Land use intensity	Abdullahi et al.^38^	Not aligned	This indicator focuses on spatial intensification and densification, which is currently misaligned with the regional profile. The Nusantara agglomeration is characterized by dispersed small-to-medium cities and large-scale greenfield expansion.	Not aligned

a These indicators are collectively utilized in the cited literature.

To achieve broad-scale sustainability, urban growth must reconcile the binary trade-off between ecological protection and economic development, as overemphasizing one invariably degrades either environmental integrity or urban viability.^83^ While the Forest City strategy currently centers on the Nusantara boundary, extending this vision to the wider agglomeration requires metrics that capture the interplay between urban systems and ecological limits. The cyclical adaptive development approach can effectively evaluate regional capacities by integrating environmental quality, conflict potential, and land suitability to delineate zones for development, reorganization, and preservation.^81^ Within this framework, indicators such as ERQ, LUCP, and UDLS collectively quantify the friction between urban dynamics and environmental constraints, clarifying complex spatiotemporal LUC and human-environment relationships.^86^ Rather than merely prohibiting conversion, these preventive measures translate sustainability functions and land-use conflicts into spatially differentiated measures of carrying capacity and development risk. This approach identifies where growth may generate cumulative ecological stress, allowing models to moderate expansion in simulation. Consequently, it aligns with the Nusantara Masterplan’s vision of balancing ecosystem protection with responsible economic utilization.

Urban climate resilience constitutes another considerable challenge for future urbanization in metropolitan Nusantara, as the concentration of development within riverine and coastal zones in Balikpapan, Samarinda, PPU, and Kukar exacerbates climate vulnerability. Empirical research confirms multi-level flood risks across subdistricts,^92^ cities,^93^ watersheds,^94^ and regional scales,^20^ necessitating a harmonized Sponge City strategy. Although traditional distance-based exclusions and policies impose spatial limits, they fail to capture the nuanced tensions between population pressure and flood vulnerability.^89^ Addressing this gap, integrating SCA indicators into LUC models enables a more realistic mapping of land suitability by linking land dynamics with rain-flood resilience.^88^^,^^89^ SCA adjusts conversion weights based on actual water-management capacity rather than static exclusion rules. This approach significantly improves the representation of hydrological constraints, ensuring that urban development remains sensitive to regional climate adaptability.^88^

Furthermore, disparities in infrastructure and economic opportunity drive uneven urbanization within the Nusantara agglomeration.^58^^,^^59^ The economic dominance of Samarinda and Balikpapan creates a stark contrast with adjacent municipalities, manifesting in divergent urbanization rates that underscore spatial inequality as a critical challenge.^95^ The Nusantara Development Masterplan, however, lacks robust mechanisms to address these disparities, raising concerns that the proposed economic superhub may exacerbate regional divides. While conventional LUC models manage inequality through localized zoning, they often neglect the inter-regional linkages that drive such imbalances. To overcome this, integrating SDH and UGI indicators provides a more accurate representation of regional interdependencies in urbanization assessment.^66^^,^^91^ In simulations, these indicators function as weighting modifiers that penalize expansion pathways likely to intensify inequality, thereby promoting land management strategies that favor more harmonious regional development.

### Predictive-preventive integration for future urbanization assessment

**Figure 7:**
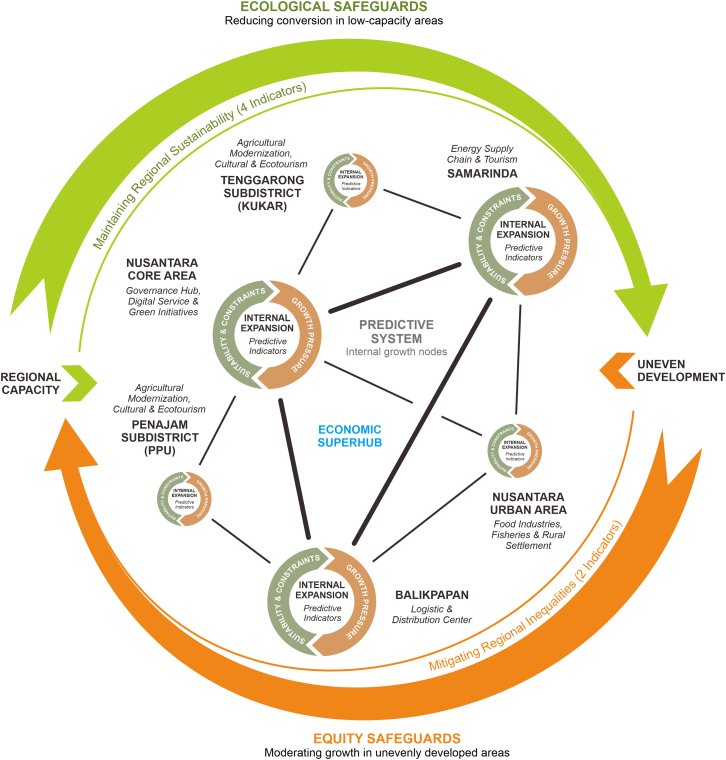
Predictive-preventive integration framework for future urbanization in the Nusantara agglomeration The city nodes and their respective economic functions represent internal growth momentum generated by the predictive system (Figure 6). Node size denotes the urban hierarchy, while connector thickness indicates the intensity of regional accessibility and spatial interaction. The green arc symbolizes ecological safeguards that mitigate conversion in low-capacity zones based on regional capacity. The orange arc represents equity safeguards that moderate growth in unevenly developed areas, addressing socio-spatial disparities across the agglomeration. The framework illustrates how internal urban expansion is adjusted by regional sustainability and equity considerations beyond administrative boundaries.

The preceding discussion demonstrates that conventional, internally driven urban models cannot adequately capture the regional ecological limits, hydrological vulnerabilities, and spatial inequalities that characterize the broader agglomeration scale. Because local socioeconomic dynamics and environmental thresholds routinely cross formal planning jurisdictions, emerging urbanization faces distinct cross-boundary externalities and sustainability risks that demand a preventive analytical lens. Building on this rationale, LUC assessments require modeling workflows that transcend isolated urban boundaries. Synthesizing the indicator evaluations in Tables 2 and 3, this study proposes an integrated schematic framework in Figure 7 that addresses these conventional gaps by linking nodal growth dynamics with cross-boundary socio-ecological constraints.

The model treats predictive indicators as internal driving forces—modeling growth within administrative boundaries, primary economic engines, and the superhub corridor. Preventive indicators, in contrast, couple this growth with broader regional interaction processes by regulating transboundary expansion pressures. This dual structure ensures that internal growth pressure is assessed alongside regional carrying capacity and socio-spatial thresholds.

In practical LUC simulations, this integration can be translated into a two-step modeling logic. First, a set of 46 predictive determinants describing internal urban conditions collectively generate a spatial attractiveness field reflecting the distribution of land suitability, centrality, and market-driven demand—assigning baseline transition probabilities for urban conversion. Second, six preventive safeguards, gauging regional environmental capacity and spatial inequality, are incorporated as resistance layers, threshold constraints, or weighting modifiers. These preventive indicators regulate rather than merely project urban expansion by modulating conversion probabilities in areas with high ecological fragility, hydrological risk, or pronounced spatial imbalance. Consequently, a location with high accessibility and development pressure may still receive a lower final transition probability if preventive indicators identify significant land-use conflict or limited environmental capacity. This dynamic interaction between attraction (predictive) and resistance (preventive) effectively decodes the spatial logic of future urbanization.

While this predictive-preventive interaction establishes a robust simulation baseline, future urban management must align these spatial indicators with emerging smart governance capabilities to operationalize this spatial-methodological framework effectively. Integrating this contextualized framework into Nusantara’s localized digital governance blueprints—such as the sustainable ICT architectures discussed by Sunindyo et al.^96^—could support transparent and continuous monitoring of cross-boundary risks. By leveraging these sustainable ICT frameworks, local government agencies can transition from reactive zoning enforcement to proactive, data-driven urban management. Specifically, predictive indicators could be updated through information on land development, accessibility, infrastructure provision, and service distribution, while preventive indicators could support continuous monitoring of ecological capacity, hydrological risk, regional vulnerability, and uneven development. This linkage clarifies how smart governance can serve as an enabling infrastructure for calibrating and monitoring predictive-preventive LUC assessment in future empirical applications.

Before such operational use, the framework necessitates rigorous calibration to avoid redundancy where indicators conceptually intersect. For instance, UDLS proxies may overlap with landscape character factors in the GP category, while UGI inherently incorporates accessibility components found in the UF category. Distinctly defining these analytical roles is essential to ensure each indicator contributes uniquely to urban growth dynamics. Consequently, future empirical applications should address redundancy through staged screening procedures. Potentially overlapping predictors can be evaluated through association-based tests, such as Cramer’s V^64^^,^^97^ and Pearson’s Correlation,^54^^,^^98^ or multicollinearity diagnostics using variance inflation factor (VIF).^48^^,^^55^ Where several indicators capture overlapping processes, dimensional-reduction techniques like principal component analysis (PCA) can synthesize composite variables while maintaining the integrity of the predictor set.^99^^,^^100^ Furthermore, sensitivity-based testing is vital to verify whether model outputs remain robust when potentially redundant indicators are excluded or reweighted.^45^^,^^54^ This workflow would confirm the framework’s practical applicability and statistical robustness in future LUC simulations.

Although contextualized for the Nusantara agglomeration, the framework’s analytical structure is adaptable to other NCDs globally. Its underlying mechanism balances internal urbanization drivers with preventive sustainability dimensions across NCD contexts.^31^ As supported by broader LUC literature, while predictive indicators are typically organized around internal drivers of land transformation, preventive safeguards can be tailored to reflect region-specific challenges, interactions, capabilities, and development visions. This adaptability ensures that the framework serves as a flexible analytical schema rather than a static indicator list.^31^ Other emerging city projects may retain this predictive-preventive logic while recalibrating the indicator composition to align with specific development paradigms, such as smart-city governance, climate-resilient urbanism, or nature-based planning. This flexibility enables robust comparative analysis and scenario modeling across diverse emerging city ecosystems. Ultimately, it safeguards a balance between development ambitions and regional capacity and equity to guide a more responsible global urban trajectory.

### Limitations of the study

Despite fulfilling its primary objectives, this study has limitations that provide avenues for future research. First, the exclusive reliance on the Scopus database, while ensuring high-quality sourcing, inevitably narrows the bibliographic scope. Second, the current framework is interpretive and conceptual. While theoretically robust, it should be viewed as a foundational template for future comparative studies rather than a finalized operational tool. Subsequent research must employ empirical validation, scenario-based LUC modeling, spatial simulation workflows, and calibration against observed land-use transitions to evaluate the framework’s predictive capability and practical usefulness in real-world urbanization contexts. Such empirical implementation will also require redundancy reduction and sensitivity testing to ensure that predictive and preventive dimensions remain analytically distinct and robust. This validation process will strengthen the translation of these scientific findings into actionable technical strategies for urbanization control.

Beyond methodology, the functional role of preventive indicators in translating regional imbalance into spatial outcomes in LUC modeling requires deeper investigation. Future research must clarify how regional capacities and urban interactions influence actual urbanization flows, particularly regarding the Forest-Sponge-Smart City vision. Extending Forest City principles beyond Nusantara’s administrative boundaries necessitates a closer examination of the friction between expansion and ecologically sensitive zones. Likewise, as the application of Sponge-Smart City strategies may yield uneven spatial results, targeted studies should explore how disparities in water-management capacity and technological infrastructure interact under contemporary growth pressures.

In response to the emerging dynamics of the Nusantara agglomeration, three priorities define the immediate research agenda. First, multi-scalar calibration is essential to capture the simultaneous unfolding of urbanization at the core, corridor, and regional levels. Second, integrating cross-boundary governance into LUC assessments is critical for managing infrastructure and interactions that exceed formal planning jurisdictions. Finally, empirical validation within the early stages of NCD will be vital to account for the unique uncertainties inherent in evolving urban functions and institutional structures. Addressing these priorities will solidify the framework’s utility as a tool for guiding sustainable urbanization in the Nusantara agglomeration.

## Conclusion

This study establishes a contextualized predictive-preventive framework of LUC indicators tailored to the unique urbanization trajectory of the Nusantara agglomeration. By synthesizing recent global literature with the Nusantara Development Masterplan, the research demonstrates that future urban expansion must be navigated through two interrelated dimensions: internal growth drivers and regional socio-ecological constraints.

Concerning RQ1, the study identifies 46 predictive indicators pertinent to the internal urban dynamics of the region’s municipalities. While GP, CC, and restrictive IP indicators define the biophysical limits of expansion, ED, UF, and incentive-based IP components serve as the primary catalysts for urban growth. Addressing RQ2, the analysis suggests that while the Forest-Sponge-Smart City vision offers a robust foundation for internal resilience, it remains insufficient for managing complex regional interactions beyond the capital’s administrative borders. To address this, the study introduces six preventive indicators—ERQ, LUCP, UDLS, SCA, SDH, and UGI—as complementary safeguards to integrate environmental sustainability and regional inequality into the broader LUC assessment across the Nusantara agglomeration.

The central contribution of the study is an integrated framework that bridges predictive determinants of localized growth with preventive indicators of regional capacity and uneven development. This approach extends LUC assessment beyond traditional boundaries, implying that achieving sustainable urbanization in Nusantara will depend less on internal planning ambitions and more on the governance of interregional interactions in a manner that is both socially inclusive and ecologically restorative. Beyond Nusantara, the framework offers an adaptable analytical structure for other NCDs. Its transferability lies in recalibrating predictive indicators to local growth drivers and adjusting preventive indicators to locally relevant spatial challenges and development visions, rather than applying the Nusantara indicator set as a fixed template.

## Acknowledgments

The authors express gratitude to the Indonesia Endowment Fund for Education (LPDP) for financial support of this work, grant no. 0008686/ESC/D/ASN-2022. The authors also thank the TUM University Library for funding the open-access publication costs through their institutional waiver program. Finally, the authors appreciate the constructive comments of the reviewers and editor, which helped improve the manuscript.

## Author contributions

Conceptualization, A.G. and W.d.V.; data curation, A.G.; methodology, A.G.; investigation, A.G. and W.d.V.; formal analysis, A.G.; software, A.G.; visualization, A.G.; writing – original draft, A.G.; validation, W.d.V.; writing – review and editing, A.G. and W.d.V.; funding acquisition, A.G.; resources, A.G. and W.d.V.; supervision, W.d.V.

## Declaration of interests

The authors declare that they have no known competing financial interests or personal relationships that could have appeared to influence the work reported in this paper.
