# Supplementary material for: Decoding urbanization pathways: An integrative land use change indicator framework for sustainable development in Nusantara
Source: iScience. 2026 Jul 9;29(8):116763. doi: 10.1016/j.isci.2026.116763 (PMC13380739; doi:10.1016/j.isci.2026.116763)
Supplement: Document S1. Tables S1–S3 [file mmc1.pdf]

iScience, Volume *29*

Supplemental information

Decoding urbanization pathways: An integrative land use change indicator framework for sustainable development in Nusantara

Achmad Ghozali and Walter Timo de Vries

iScience, Volume X

Supplemental Document

Decoding urbanization pathways: An integrative land use change indicator framework for sustainable development in Nusantara

Achmad Ghozali, Walter Timo de Vries

Supplemental Contents

Table S1. Key Features and New Infrastructure of Transformation Development Concepts in the Nusantara Development Masterplan 3

Table S2. Contextualization of Constraining LUC indicators found in the collected literature in the Nusantara agglomeration context 5

Table S3. Contextualization of Driving LUC indicators found in the collected literature in the Nusantara agglomeration context 7

Supplemental references 11

Table S1. Key Features and New Infrastructure of Transformation Development Concepts in the Nusantara Development Masterplan

Concept	Core Idea	Main Strategies	New Planned Infrastructure	Urbanization Influence
Forest City	Preserve the ecological backbone by maintaining ≥65% of Nusantara areas as protected and restored forest	▪ 100,000 ha conservation (primary forest, secondary forest, and mangrove)<br>▪ 40,000 ha agroforestry/sustainable industrial plantation forest<br>▪ 30,000 ha restoration and wildlife corridors<br>▪ 29,000 ha urban green (city forests, parks, and vertical gardens)	▪ Urban ecological infrastructure: city forests, botanical gardens, thematic parks, riparian green corridors, and vertical/roof gardens<br>▪ Conservation facilities: wildlife corridors, conservation centers, and biodiversity parks<br>▪ Reforestation infrastructure: large-scale nurseries, degraded land reclamation (ex-mining/logging)<br>▪ Community-based facilities: eco-tourism nodes, agroforestry demonstration zones	▪ Directional development by limiting sprawl<br>▪ Biodiversity protection and carbon sequestration<br>▪ Climate resilience through ecological buffers<br>▪ Diversified land economy via eco-services
Sponge City	Restore natural hydrological cycles, making the city function like a sponge	▪ Archipelago City: green and blue structure<br>▪ Absorbent City: corridors, wetlands, riparian parks<br>▪ Integrated City: green roofs, permeable pavements, retention basins	▪ Water supply: Sepaku-Semoi Dam, river intakes, multipurpose reservoirs, main pipelines, wastewater treatment plants, storage reservoirs, and water pipeline networks<br>▪ Flood control and optimal drainage: drainage channels, sediment control, retention basins, wetlands, and floodplain zones<br>▪ Urban water-sensitive features: rainwater harvesting systems, infiltration wells, permeable pavements, green roofs, and vertical greenery<br>▪ Climate adaptation infrastructure: interconnected lakes, sponge parks, and bio-retention swales	▪ Reduced flood risk and runoff<br>▪ Groundwater recharge to improve water supply<br>▪ Cooling and reduced UHI<br>▪ Water-sensitive development and protection of low-lying areas

Smart City	Digital governance and ICT to enhance efficiency, inclusivity, and resilience	Six initiative smart clusters:<br>Smart urban systems<br>1. Safety and security<br>2. Digital governance<br>3. Environmental monitoring<br>4. Smart mobility<br>5. Liveability & culture	▪ Digital backbone: fiber optic networks (100–400 GbE), multi-ring network, 100–200 BTS, edge data centers, and national integrated data center<br>▪ Smart governance platforms: e-citizen portals, integrated databases, digital licensing/tax systems, and participatory apps<br>▪ Urban systems: smart grids, smart water meters, waste-to-energy facilities, environmental monitoring dashboards<br>▪ Safety and resilience: smart lighting, disaster early-warning systems, emergency management centers<br>▪ Smart mobility: Bus Rapid Transit (BRT) corridors, TOD hubs, electric buses and charging stations, real-time parking, integrated transport apps, MRT/LRT plan<br>▪ Liveability: e-health platforms, smart housing, e-learning systems, and digital culture/tourism archives	▪ Optimized infrastructure and reduced congestion<br>▪ Transparent and participatory governance<br>▪ Equitable access to services<br>Innovation-driven economy supporting inclusive urbanization

Table S2. Contextualization of Constraining LUC indicators found in the collected literature in the Nusantara agglomeration context

Indicators	Measurement	Occurrences	Relevancy	Contextual Relevance	Supporting reference
PG Category					
Elevation	Continuous raster	105	A	Existing urban centers concentrate in more developable terrain near the coast	Yudhantoro et al.[1]
Slope	Continuous raster	113	A	The region has a landslide risk due to the terrain conditions; Steep terrain limits expansion	Yudhantoro et al.[1]
Aspect	Continuous raster	27	A	Affect the microclimate that is relevant to settlement comfort	Li et al.[2*]
Soil type	Categorical soil	25	A	Soil texture determines construction stability	Abdullahi & Pradhana[3*]
Geological properties	Categorical rock	4	A	Determines soil and construction stability	Wang et al.[4]
Faults	Proximity	2	X	Active faults lie outside the Nusantara region and have low seismic risk	Nur Azhar et al.,[5]
General water bodies	Proximity, Exclusion Area	34	S	Align with rivers	
Rivers	Proximity, Exclusion Area	33	A	Existing city development follows an aquatic system, such as a river estuary and coastal morphology	Yudhantoro et al.[1]
Coastal	Proximity	7	A	Align with rivers	
Highly vegetated area	Proximity; Exclusion Area	4	A	The regions surrounded by protected and production forests, which have been considered for their development	Spencer et al.[6*]
NDVI	Index	3	A	It indicates vegetation density and ecosystem vitality	Li et al.[2*]
CE Category					
Precipitation	Continuous raster	36	A	Rainfall trends in the Nusantara agglomeration region are expected to increase the risk of geological disasters.	Ramadhan et al.[7]
Temperature	Continuous raster	33	A	Urban development across the Nusantara agglomeration region faces the challenge of urban	Rushayati et al.,[8] Pradana et al.[9]

				heat due to massive development and forest encroachment.	
Evaporation	Continuous raster	3	A	Reflects climatic stress on land productivity and surface water dynamics	An et al.[10]*
Solar radiation	Continuous raster	2	A	Determines energy potential and contributes to surface temperature variation	An et al.[10]*
Flood zone	Proximity, Exclusion area	6	A	Many studies confirmed that flood disasters affect the development of cities around the Nusantara	Spencer et al.,[6] Heo et al.[11]
PR Category - Repressive					
Ecological land preservation zone	Exclusion area	28	A	Existing protected forests are included as natural conservation areas and restoration forests in the IKN Nusantara Masterplan, which limits urban expansion	Arfiansyah et al.[12]
Farmland preservation zone	Exclusion area	12	A	There are protected paddy fields in East Kalimantan, distributed across Kutai Kartanegara, Balikpapan, Samarinda, and Penajam Paser Utara Regencies. This decision affects rice field conservation zones that are not subject to conversion	Chairunnisya & Jamil,[13]
Historical preservation zone	Exclusion area	2	A	Existing cities have policies related to protected historical sites, typically related to the Kutai Kingdom legacy and Dayak cultures	Alfaqi et al.,[14] Lirim et al.[15]

A = Applicable (directly relevant in the Nusantara context); X = Not applicable (irrelevant or unavailable in the current spatial context); S = Substitutable (the indicator's effect is already represented by another correlated or proxy); * = Generalizability (the contextualization comes from general LUC research

Table S3. Contextualization of Driving LUC indicators found in the collected literature in the Nusantara agglomeration context

Indicator	Defined Urban Features	Measurement	Occurrences	Relevancy	Contextual Relevance	Supporting reference
SE Category						
Total Population	-	Statistical attribute	83	A	Population density strongly correlates with future built-up land demands, often extending development into less suitable lands for construction	Virtriana et al.[16]
Urbanization rate	-	Index (Boundary-based)	2	A	The varying fiscal capacities of municipalities form development disparity, signifying the intensity of urbanization	Espinosa et al.,[18] Lestari et al.[17]
Migration rate	-	Statistical attribute	4	A	Nusantara establishment promotes migration, which will reinforce the population and alter green landscapes	Syaban & Appiah-Opoku,[19]
GDP	-	Statistical attribute	52	A	Variations in regional GDP (GRDP) are evident across regions, affecting investment and infrastructure development.	Yusuf et al.[20]
Night light intensity	-	Continuous raster	13	A	Serves as a proxy for economic activity and urban concentration	Subagio et al.[21]
Employment rate	-	Statistical attribute	3	A	Workforce availability promotes regional economic growth and urban expansion	Putra et al.[22]
Employment in service sector	-	Statistical attribute	3	A	Capital relocation accelerates massive structural change into relatively high-skill intensive sectors, increasing land development	Putra et al.,[22] Andriansyah et al.[23]
Employment in the secondary sector	-	Statistical attribute	2	A	Despite economic shifts driven by Nusantara, the natural resource-based manufactures maintain their productivity and function as a catalyst for growth	Putra et al.,[22] Andriansyah et al.[23]
Land value	-	Statistical attribute	4	A	Nusantara development escalates land values in and around the new capital	Agustino et al.[24]
Secondary industrial outputs	-	Statistical attribute	2	A	Manufacturing productivity remains an economic cornerstone of the municipalities surrounding Nusantara	Andriansyah et al.[23]

Investment in infrastructure	-	Statistical attribute	2	X	Concentrated mainly in the Nusantara core areas, it has a limited reflection of broader regional change	Nusantara Development Masterplan[26]
UA Category- Centrality						
Economic center	CBD, city-, district-, sub-district center	Proximity	57	A	Existing economic cores typically revolve around the government district, commonly promoting a trade and services hub	Zhao et al.[25*]
General urban land use	built-up, urbanized areas	Proximity, Density	45	A	Reflecting the area concentration of urban infrastructure and accessibility	Jamali & Ghorbani Kalkhajeh,[27*]
Specific urban land use	Industrial, urban residential/real estate, vacant land	Proximity	21	A	The rise of industrial, mining, and new residential areas, particularly in Balikpapan, Samarinda, and PPU, strongly promotes economic growth and drives LUC	Syaban & Appiah-Opoku,[28]
Specific rural land use	agricultural, mining, livestock	Proximity, Density	9	A	Aligns with the contextualization in the specific urban land use indicator; only mining areas promote spatial transformation in East Kalimantan	Syaban & Appiah-Opoku,[28]
UA Category - Amenities						
General urban facilities	Urban point of interest	Proximity, Density	9	S	Few LUC studies use a general measure. The effect of different types of urban facilities on LUC is crucial for developing specific land management strategies.	
Educational facility	Elementary, secondary, and tertiary schools	Proximity, Density	20	A	Positively correlated with rural development in East Kalimantan	Wiratama et al.[29]
Medical facility	Hospital, Health-care center	Proximity	18	A	Positively correlated with rural development in East Kalimantan	Wiratama et al.[29]
Commercial site	Restaurant, shopping center, market, mall, supermarket, commercial building	Proximity, Density	9	A	Positively correlated with rural development in East Kalimantan	Wiratama et al.[29]

Public space facility	Park, plaza	Proximity	10	A	Positively correlated with rural development in East Kalimantan	Wiratama et al.[29]
Public safety facility	Police station, Military office	Proximity	4	A	It contributes to the perceived urban quality of life	Z. Wang et al.[30]*
Workplace facility	Government office, office building, bank	Proximity	11	S	In small and medium-sized cities, government and trade services-based offices are primarily dominated which is adequately represented by the urban economic centre factor	
Leisure and community facility	Community building, gallery, museum, entertainment building, stadium	Proximity	6	X	As dominated by small and medium-sized cities, these facilities are not a primary need for the general public.	
Tourism site	Tourism attraction area	Proximity	10	A	The tourism industry exhibits an increasing trend in East Kalimantan, which drives economic development	Saha Ghafur,[31]
Utility management sites	Sanitary building, water service building, electricity center, waste treatment plant	Proximity	5	A	Positively correlated with rural development in East Kalimantan	Wiratama et al.[29]
UA Category – Mobility						
General transportation nodes	Transport interchange	Proximity	3	S	Aligns with indicators related to specific transport hubs	
Air transport	Airport	Proximity	10	A	The capital's relocation to East Kalimantan was driven by higher regional accessibility, particularly the presence of airports, which are vital for transit-oriented development.	Nur Azhar et al.[5]
Water transport	Port	Proximity	4	A	Existing urban centers have grown into coastal cities with ports as essential	Yudhantoro et al.[1]

					transportation hubs such as Balikpapan and Penajam Paser Utara	
Bus transit	Terminal, sub-terminal	Proximity	10	A	It stimulates transit-oriented expansion	Zhao et al.[25]*
Road transit	Highway entrance, toll gate	Proximity	3	A	It stimulates transit-oriented expansion	Zhao et al.[25]*
Rail transit	Rail station, metro/subway station	Proximity	24	X	No railway infrastructure currently exists in East Kalimantan	
General roads	Roads	Proximity	58	S	Aligns with specific functional level roads	
Highways	Highway, expressway, toll road	Proximity	45	A	Balikpapan-Samarinda toll roads play a significant role in restructuring the regional economy	Tarassyta & Auwalin,[32]
Railways	Railroads, subways	Proximity	51	X	No railway infrastructure currently exists in East Kalimantan	
Functional level roads	Primary, secondary, and tertiary roads	Proximity	20	A	Road functionalities in East Kalimantan's impact on traffic efficiency and regional economic growth	Kurniawan et al.[33]
Regional hierarchy roads	National, provincial, county, and local roads	Proximity	15	S	Aligns with specific functional level roads	
Road load capacity	First-, second-, third-, fourth-class road	Proximity	3	S	Aligns with specific functional level roads	
Service priority roads	Major, minor road	Proximity	26	S	Aligns with specific functional level roads	
PR Category – Catalyst						
Urban land plan	New urban development, special economic, political center,	Proximity, Boundary	13	A	The IKN Nusantara development Masterplan mandates several new urban development zones that have great potential to be converted into built-up land	Nusantara Development Masterplan[26]

	infrastructure plan					
Transportation plan	Highways, road network, railways, train station plan	Proximity	6	A	Several designated road networks are to be developed, including toll roads, railway systems, and local roads, which will connect the IKN Nusantara region with its vicinity	Nusantara Development Masterplan[26]

A = Applicable (directly relevant in the Nusantara context); X = Not applicable (irrelevant or unavailable in the current spatial context); S = Substitutable (the indicator's effect is already represented by another correlated or proxy); * = Generalizability (the contextualization comes from general LUC research

Supplemental references

1. Yudhantoro, W., Januari, A., Utami M., A., Pitaloka, E., Indah A., K., Rusdayanti, N., Damayanti, P., Wardana, S., Pramiati, S., Shara, S., et al. (2020). Implementation of Spatial and Development Planning in East Kalimantan Province. In Proceedings of the Proceedings of the 1st International Conference on Environmental Science and Sustainable Development, ICESSD 2019, 22-23 October 2019, Jakarta, Indonesia (EAI). https://doi.org/10.4108/eai.22-10-2019.2291496.
2. Li, C., Huang, J., Luo, Y., and Wang, J. (2025). Spatial Synergy Between Carbon Storage and Emissions in Coastal China: Insights from PLUS-InVEST and OPGD Models. Remote Sens. (Basel). *17*, 2859. https://doi.org/10.3390/rs17162859.
3. Abdullahi, S., and Pradhan, B. (2018). Land use change modeling and the effect of compact city paradigms: integration of GIS-based cellular automata and weights-of-evidence techniques. Environ. Earth Sci. *77*, 251. https://doi.org/10.1007/s12665-018-7429-z.
4. Wang, L., Pijanowski, B., Yang, W., Zhai, R., Omrani, H., and Li, K. (2018). Predicting multiple land use transitions under rapid urbanization and implications for land management and urban planning: The case of Zhanggong District in central China. Habitat Int. *82*, 48–61. https://doi.org/10.1016/j.habitatint.2018.08.007.
5. Nur Azhar, H., Putri Fatima, H.H., and Tamas, I.N. (2020). Preliminary study of indonesia capital city relocation based on disaster mitigation principle with mental model approach. E3S Web of Conferences *148*, 06002. https://doi.org/10.1051/e3sconf/202014806002.
6. Spencer, K.L., Deere, N.J., Aini, M., Avriandy, R., Campbell-Smith, G., Cheyne, S.M., Gaveau, D.L.A., Humle, T., Hutabarat, J., Loken, B., et al. (2023). Implications of large-scale infrastructure development for biodiversity in Indonesian Borneo. Science of The Total Environment *866*, 161075. https://doi.org/10.1016/j.scitotenv.2022.161075.
7. Ramadhan, R., Marzuki, M., Suryanto, W., Sholihun, S., Yusnaini, H., Muharsyah, R., and Hanif, M. (2022). Trends in rainfall and hydrometeorological disasters in new capital city of Indonesia from long-term satellite-based precipitation products. Remote Sens. Appl. *28*, 100827. https://doi.org/10.1016/j.rsase.2022.100827.
8. Rushayati, S.B., Prasetyo, L.B., Pramesti, T.V., Wijaya, F.H., and Wijayanto, A.K. (2025). Spatio-temporal analysis of land surface temperature and biomass changes in Nusantara Capital City: Challenges for forest city planning. Ecological Engineering & Environmental Technology *26*, 74–85. https://doi.org/10.12912/27197050/210101.

9. Pradana, R.P., Bhanage, V., Fajary, F.R., Hussainzada, W., Badriana, M.R., Lee, H.S., Kubota, T., Nimiya, H., and Putra, I.D.G.A. (2025). Assessing Green Strategies for Urban Cooling in the Development of Nusantara Capital City, Indonesia. Climate *13*, 30. https://doi.org/10.3390/cli13020030.

10. An, X., Zhang, M., and Zang, Z. (2023). Driving Mechanisms of Spatiotemporal Heterogeneity of Land Use Conflicts and Simulation under Multiple Scenarios in Dongting Lake Area. Remote Sens. (Basel). *15*. https://doi.org/10.3390/rs15184524.

11. Heo, S., Sohn, W., Park, S., and Lee, D.K. (2024). Multi-hazard assessment for flood and Landslide risk in Kalimantan and Sumatra: Implications for Nusantara, Indonesia's new capital. Heliyon *10*, e37789. https://doi.org/10.1016/j.heliyon.2024.e37789.

12. Arfiansyah, D., Hawken, S., Zlatanova, S., and Han, H. (2024). Cellular automata modelling to simulate patterns of urban growth for Nusantara: Indonesia's new capital. Spatial Information Research *32*, 829–849. https://doi.org/10.1007/s41324-024-00599-5.

13. Chairunnisya, R.A., and Jamil, A. (2024). Developing food security with sustainable agricultural land policies: A systematic review. E3S Web of Conferences *479*, 07022. https://doi.org/10.1051/e3sconf/202447907022.

14. Alfaqi, M.Z., Shofa, Abd.M.A., Mawarti, R.A., Pratama, A.Y., Azizah, R.S.N., Abadi, D.P., and Agung, L.P. (2023). Ethnocultural Exploration: Ethnic Cultural Study of East Kalimantan in the National Capital Relocation as a Cultural Resilience Strategy. In, pp. 189–199. https://doi.org/10.2991/978-2-38476-168-5_18.

15. Lirim, A., Habibi, M., Eryani, T.W.R., and Pratama, P.Y. (2025). Preserving Cultural Heritage Through Traditional Institutions and Youth Engagement. Academia Open *10*. https://doi.org/10.21070/acopen.10.2025.11419.

16. Virtriana, R., Ihsan, K.T.N., Anggraini, T.S., Harto, A.B., Riqqi, A., and Deliar, A. (2025). Predicting Suitable Built-up Areas in Indonesia's New Capital: Integrating Ecosystems, Access, and Socioeconomics. Sustainable Futures *10*, 101342. https://doi.org/10.1016/j.sftr.2025.101342.

17. Lestari, R.I., Wardono, B., Handajani, M., Supari, S., Juniati, H., Sunarno, M.T.D., and Prayogi, E. (2025). The interplay of road infrastructure and regional finance in driving economic growth: Insights from East Kalimantan. Journal of Open Innovation: Technology, Market, and Complexity *11*, 100444. https://doi.org/10.1016/j.joitmc.2024.100444.

18. Espinosa, Y.G., Suparta, I.W., and Moniyana, R. (2024). Income Disparities and Regional Economic Potential in East Kalimantan Province as the National Capital (IKN) New Capital City of Indonesia Nusantara. International Journal of Economics, Management and Accounting (IJEMA) *2*, 43–54. https://doi.org/10.47353/ijema.v2i1.144.

19. Syaban, A.S.N., and Appiah-Opoku, S. (2023). Building Indonesia's new capital city: an in-depth analysis of prospects and challenges from current capital city of Jakarta to Kalimantan. Urban Plan. Transp. Res. *11*. https://doi.org/10.1080/21650020.2023.2276415.

20. Yusuf, A.A., Roos, E.L., Horridge, J.M., and Hartono, D. (2023). Indonesian capital city relocation and regional economy's transition toward less carbon-intensive economy: An inter-regional CGE analysis. Japan World Econ. *68*, 101212. https://doi.org/10.1016/j.japwor.2023.101212.

21. Subagio, Nainggolan, T.B., Kusnida, D., Mirnanda, E., Ervan, M., and Setyanta, B. (2025). Gravity mapping and subsurface geologic analysis for the new capital of Indonesia: insights into the Penajam Paser Utara Regency. Geosciences Journal *29*, 615–626. https://doi.org/10.1007/s12303-025-00035-2.

22. Putra, M., Saleh, M., and Roy, J. (2025). Analysis of Potential Economic Sectors in East Kalimantan Province and its Influence on the Superhub of the National Capital Development of the Archipelago. Jurnal Ekonomi dan Pembangunan Indonesia *3*, 231–243. https://doi.org/10.61132/jepi.v3i3.1648.

23. Andriansyah, Nurwanda, A., and Rifai, B. (2023). Structural Change and Regional Economic Growth in Indonesia. Bull. Indones. Econ. Stud. *59*, 91–117. https://doi.org/10.1080/00074918.2021.1914320.

24. Agustino, L., Hikmawan, M.D., and Silas, J. (2024). Is it possible for sustainability? The case from the new capital city of Indonesia. Front. Polit. Sci. *6*. https://doi.org/10.3389/fpos.2024.1362337.

25. Zhao, Z., Guan, D., and Du, C. (2020). Urban growth boundaries delineation coupling ecological constraints with a growth-driven model for the main urban area of Chongqing, China. GeoJournal *85*, 1115–1131. https://doi.org/10.1007/s10708-019-10014-4.

26. Peraturan Presiden Republik Indonesia Nomor 63 Tahun 2022 Tentang Perincian Rencana Induk Ibu Kota Nusantara (Presidential Regulation of The Republic of Indonesia Number 63 of 2022 Concerning The Details of The Master Plan of The Nusantara Capital City) (2022).

27. Jamali, A.A., and Ghorbani Kalkhajeh, R. (2019). Urban environmental and land cover change analysis using the scatter plot, kernel, and neural network methods. Arabian Journal of Geosciences *12*. https://doi.org/10.1007/s12517-019-4258-7.

28. Syaban, A.S.N., and Appiah-Opoku, S. (2024). Unveiling the Complexities of Land Use Transition in Indonesia's New Capital City IKN Nusantara: A Multidimensional Conflict Analysis. Land (Basel). *13*, 606. https://doi.org/10.3390/land13050606.

29. Wiratama, B.F., Kurniawan, R., Mulyanto, Isnaeni, M.A., Sumargo, B., and Gio, P.U. (2023). Measuring the physical infrastructure development as poverty reduction program in Kalimantan, Indonesia. Cities *141*, 104515. https://doi.org/10.1016/j.cities.2023.104515.

30. Wang, Z., Feng, T., Safikhani, A., and Tepe, E. (2025). Enhancing transparency in land use change modeling: Leveraging eXplainable AI techniques for urban growth prediction with spatially distributed insights. Comput. Environ. Urban Syst. *121*, 102322. https://doi.org/10.1016/j.compenvurbsys.2025.102322.

31. Saha Ghafur, A.H. (2024). Tourism policy impact on transforming religious traditions of Dayak a case study of the Hudoq ceremony tradition of the Dayak Bahau tribe in tourism development, East Kalimantan, Indonesia. Cogent Arts Humanit. *11*. https://doi.org/10.1080/23311983.2024.2429938.

32. Tarassyta, A., and Auwalin, I. (2024). The Effect of Balikpapan-Samarinda Toll Road Construction with a PPP Scheme on The Formation of Economic Structure in East Kalimantan. Jurnal Ilmu Ekonomi Terapan *9*, 51–63. https://doi.org/10.20473/jiet.v9i1.57456.

33. Kurniawan, H., Suparma, L., and Tri, U. (2024). The model for integrating road function, road status, and road class in the road network system. Journal of Applied Engineering Science *22*, 694–706. https://doi.org/10.5937/jaes0-51784.
